# Frequency response function data of a composite plate under various damage identification scenarios

**DOI:** 10.1016/j.dib.2025.111385

**Published:** 2025-02-10

**Authors:** Nathan Dwek, Dennis Janssens, Vasileios Dimopoulos, Matteo Kirchner, Elke Deckers, Frank Naets

**Affiliations:** a Department of Mechanical Engineering, KU Leuven, Celestijnenlaan 300, B-3001, Heverlee, Belgium; b Campus Diepenbeek, Department of Mechanical Engineering, KU Leuven, Wetenschapspark 27, B-3590, Diepenbeek, Belgium; c Flanders Make @ KU Leuven, Belgium

**Keywords:** Non-destructive testing, Structural health monitoring, Wave propagation, Inverse problems, Modal testing

## Abstract

This article presents data collected in support of research on damage identification in a composite plate, using low-frequency vibration measurements. The data consist of the Frequency Response Functions (FRFs) of a square plate, measured using an impact hammer and accelerometers, as an alternative to ultrasonic measurements. The plate is instrumented with 7 accelerometers, and a dense grid of candidate damage locations is defined. First, a set of baseline FRFs is collected by measuring the responses to hammer excitations at all grid points. Then, 6 damage identification scenarios are considered, and for each scenario, a set of FRFs is collected by measuring the responses to hammer excitations at the 7 accelerometer locations. Added masses are glued to the plate to reproduce the scattering effect of damage. This provides a convenient academic example for a wide range of topics in the field of structural dynamics and serves as a first proof-of-concept exercise for damage identification research. In addition, it allows to validate different approaches and benchmark their performance, thereby contributing a reference test case to adequately compare methods against one another.

Specifications Table

**Table:** 

Subject	Mechanical Engineering
Specific subject area	Damage Identification in Plate-like Structures
Type of data	Frequency Response Functions as .mat files and .npz files
Data collection	The data were collected by conducting impact testing on a composite plate with and without damage. A PCB 086C03 impact hammer with a force cell was used to hit the plate and record the exciting force, and 7 PCB 352a24 lightweight 1D-accelerometers were placed to record the resulting vibrations. Siemens Simcenter was used to acquire the data and compute the Frequency Response Functions. Measurements were averaged over 5 repetitions to reduce the impact of noise. The baseline responses were collected by hitting all 144 candidate damage locations on the healthy plate. For each subsequent inspection scenario, masses were glued to the plate to simulate damage and the responses were collected by hitting the 7 accelerometer locations.
Data source location	Department of Mechanical Engineering, KU Leuven, Celestijnenlaan 300, B-3001 Leuven, Belgium
Data accessibility	Repository name: KULeuven Research Data Repository (RDR), mirrored on Zenodo Data identification number: DOI: 10.48804/GDE9TW Direct URLs to data: 10.48804/GDE9TWhttps://zenodo.org/records/11033677 https://rdr.kuleuven.be/dataset.xhtml?persistentId=doi:10.48804/GDE9TW
Related research article	
	N. Dwek, V. Dimopoulos, D. Janssens, M. Kirchner, E. Deckers, and F. Naets, “Damage identification in plate-like structures using frequency-coupled ℓ1-based sparse estimation,” *Mechanical Systems and Signal Processing*, vol. 224, p. 112084, Feb. 2025, doi: 10.1016/j.ymssp.2024.112084. [1]

## 1 Value of the Data

• These data consist of Frequency Response Functions (FRFs) measured on a square composite plate. 7 sets of measurements are provided, each collected using an impact hammer with a force cell and 7 1D-accelerometers. These sets are organized as follows: first, a set of baseline FRFs with excitations at a dense grid of 144 candidate damage locations, and second, with different combinations of masses added to the plate, 6 sets of FRFs with excitations at the accelerometer locations.
• These measurements offer valuable insights into the vibrational behaviour the plate, including the effect of added masses, as a surrogate for the scattering effect of damage. The datasets constitute a convenient academic example for a wide range of topics in the field of structural dynamics and serve as a first proof-of-concept exercise for damage identification research.
• These data allow to validate different damage identification approaches and benchmark their detection and localization performance. This contributes a reference test case to adequately compare methods against one another.
• These data add to the list of validation cases that use added masses as a surrogate for the scattering effect of damage. As such, they provide one more comparison point to understand the fidelity and range of validity of this approximation, and to compare the vibrational properties of different types of defects and defect surrogates.
• Thanks to the high redundancy in terms of the number of excitations, sensors, and candidate damage locations, and thanks to the high frequency resolution and Signal to Noise Ratio (SNR) of the measured FRFs, these data allow to explore different configurations and degrees of difficulty in terms of sensor placement, grid density and measurement quality by discarding parts of the data and/or adding noise before processing.
• Inspection scenarios with 3 to 6 added masses, which is a larger number of defects than usually considered, allow to assess the validity of the Born approximation as the number of scatterers increases. This also presents a unique challenge to test the limits of sparsity-promoting damage identification methods such as the Lasso.

## 2 Background

These data were collected in support of research on damage identification in a composite plate, using lower-cost vibration measurements. In [1], after reviewing the relevant literature, we identified low-frequency modal testing, using an impact hammer and accelerometers, as a promising alternative to the well-established but more involved family of ultrasonics-based inspection techniques.

Our approach relies on a well-accepted working principle: defects in a structure scatter incident elastic waves in all directions, and, under the Born approximation, the scattered vibrations can be decomposed into individual contributions by each defect [2]. This requires (i) a set of FRFs from the healthy plate to establish a baseline and construct the decomposition dictionary and (ii) a smaller set of FRFs from the inspected plate, to be decomposed onto the aforementioned dictionary.

This dataset [3] was collected according to these requirements. To practically generate different inspection scenarios, masses are glued to the plate to simulate the effect of damage. This is a good surrogate for common defects [2,4,5], and has proven to be a popular validation approach [[6], [7], [8], [9]].

This dataset allowed to validate and benchmark the approach we proposed in [1], and could serve as a test case for alternative approaches, while also being relevant to structural dynamics research in general.

## 3 Data Description

The data are provided as both .mat files (to be used with MATLAB or compatible software), and as .npz files (to be used with Python or compatible software). Note that the convention of each language is respected when it comes to indexing. This means that node indexing starts at 1 in the .mat files, and at 0 in the .npz files. As a result, the node indices in the probing_nodes and mass_nodes variables are all offset by one in the .mat files compared to the .npz files. In this article, the MATLAB convention is used. The data files have descriptive names corresponding to their respective scenarios, as listed in Table 1.

**Table 1:** Inventory of data files and corresponding scenarios.

MATLAB-compatible	Python-compatible	Description
baseline.mat	baseline.npz	Plate with no added masses
		Baseline responses from all candidate damage locations to the accelerometer locations
		Auxiliary data general to all scenarios
1 mass.mat	1 mass.npz	55 g mass added at node 75
3 masses.mat	3 masses.npz	55 g masses added at nodes 20, 75, 125
4 masses.mat	4 masses.npz	55 g masses added at nodes 20, 28, 75, 125
5 masses.mat	5 masses.npz	55 g masses added at nodes 20, 28, 69, 75, 125
6 masses.mat	6 masses.npz	55 g masses added at nodes 20, 28, 69, 75, 107 125
1 elongated mass.mat	1 elongated mass.npz	315 g mass spanning nodes 81, 92, 103

Table 2 lists the data contained in the baseline file, and Table 3 lists the data contained in each data file corresponding to an inspection scenario.

**Table 2:** Contents of the baseline data file.

Variable	Type	Description
frequency	1 × 8192 double [Hz]	Bin center frequencies
node_position	144 × 2 double [mm]	Position of the candidate nodes on the plate
probing_nodes	1 × 7 uint16	Indices of the accelerometer nodes
frf	7 × 144 × 8192 double [g/N]	Baseline FRFs from all 144 candidate nodes to the 7 accelerometer nodes

**Table 3:** Contents of the data file for each inspection scenario.

Variable	Type	Description
mass_nodes	1xn uint16	Indices of the *n* nodes where masses were glued
frf	7 × 7 × 8192 double [g/N]	FRFs from the 7 accelerometer nodes to the 7 accelerometer nodes (multistatic data matrix). First index corresponds to accelerometer index, second index corresponds to excitation index.

For both MATLAB and Python, helper functions and example scripts are provided as listed in Table 4.

**Table 4:** Functions and example scripts.

Function/Script Name	Description
loader	Load baseline data, inspection data and auxiliary data for a given inspection scenario
damage_plotter	Plot a given damage map
example_plot_frf	Demonstrate plotting FRF data, practical introduction to variables and their structure
example_lsqminnorm	Demonstrate damage identification using $l_{2}$-regularization
example_music	Demonstrate damage identification using MUSIC

## 4 Experimental Design, Materials and Methods

### 4.1 Experimental setup

Data was collected from a 600 mm × 600 mm × 4 mm CFRP plate with cross-ply layup, weighing 2270 g. To index the positions of the sensors, added masses, and candidate damage nodes, a 12 × 12 square grid is defined on the plate, with 50 mm spacing and 25 mm offset from the edges. The plate is hung with elastic bands to obtain free-free boundary conditions, with the top left node denoted as node 1. This node is the origin of the coordinate system (*x* = 0 mm, *y* = 0 mm), with the X axis pointing to the right, and the Y axis pointing down.

**Fig. 1:**
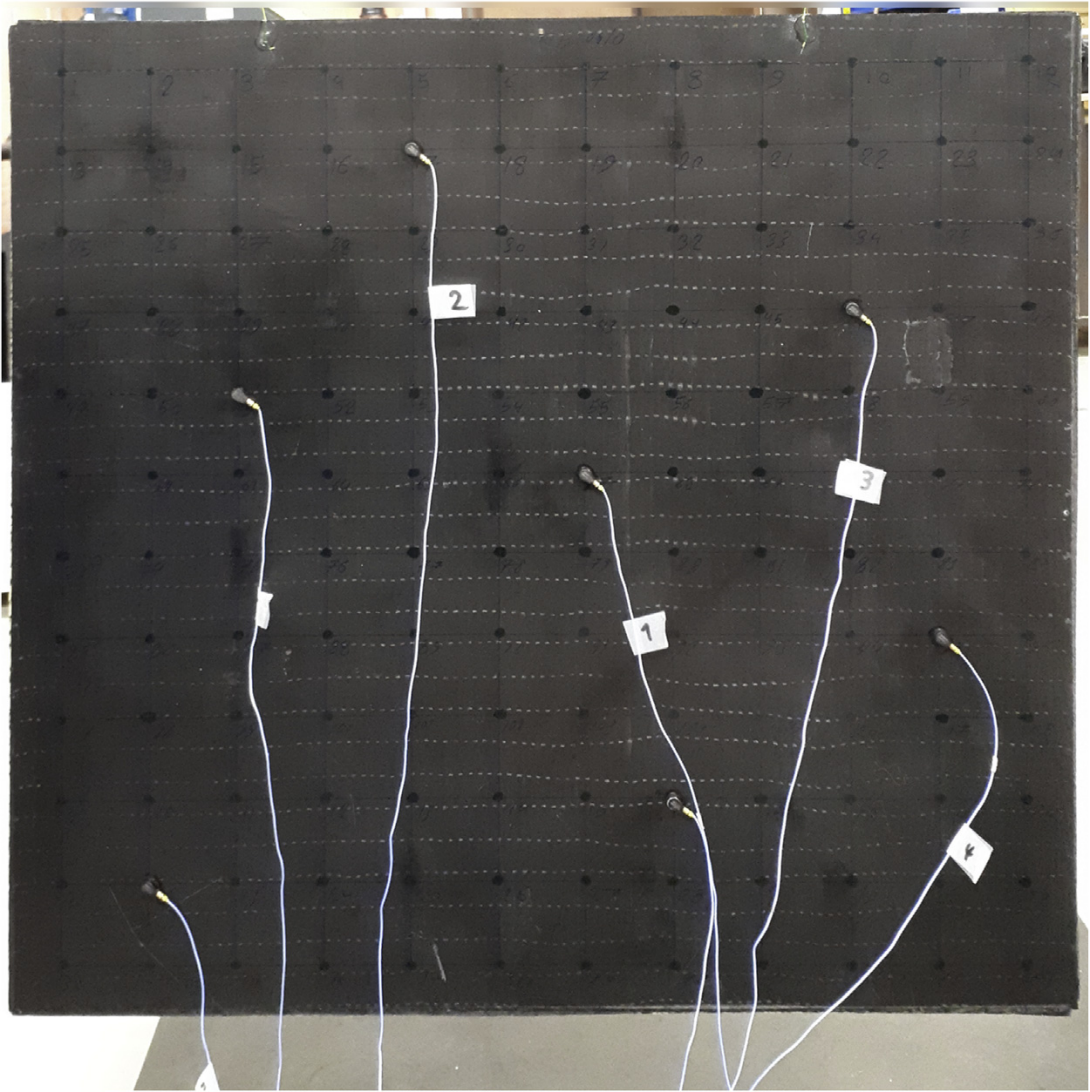
CFRP plate with 7 lightweight teardrop 1D-accelerometers attached using wax.

As shown in Fig. 1, 7 lightweight 1D-accelerometers (PCB Piezotronics, Model 352A24) are attached using wax to the plate at the positions indicated in Table 5. The plate is excited with an impact hammer (PCB Piezotronics, Model 086C03), with a medium stiffness plastic tip (PCB Piezotronics, Model 084B04, commonly denoted as “white plastic tip”). The force cell in the impact hammer measures the excitation signal, which, together with the accelerometer measurements, are used to compute the corresponding FRFs.

**Table 5:** Accelerometer/Excitation nodes.

Accelerometer index *j*	1	2	3	4	5	6	7
**Node index *i***	17	46	51	67	95	116	122
***x* [mm]**	200	450	100	300	500	350	50
***y* [mm]**	50	150	200	250	350	450	500

### 4.2 Inspection scenarios

**Fig. 2:**
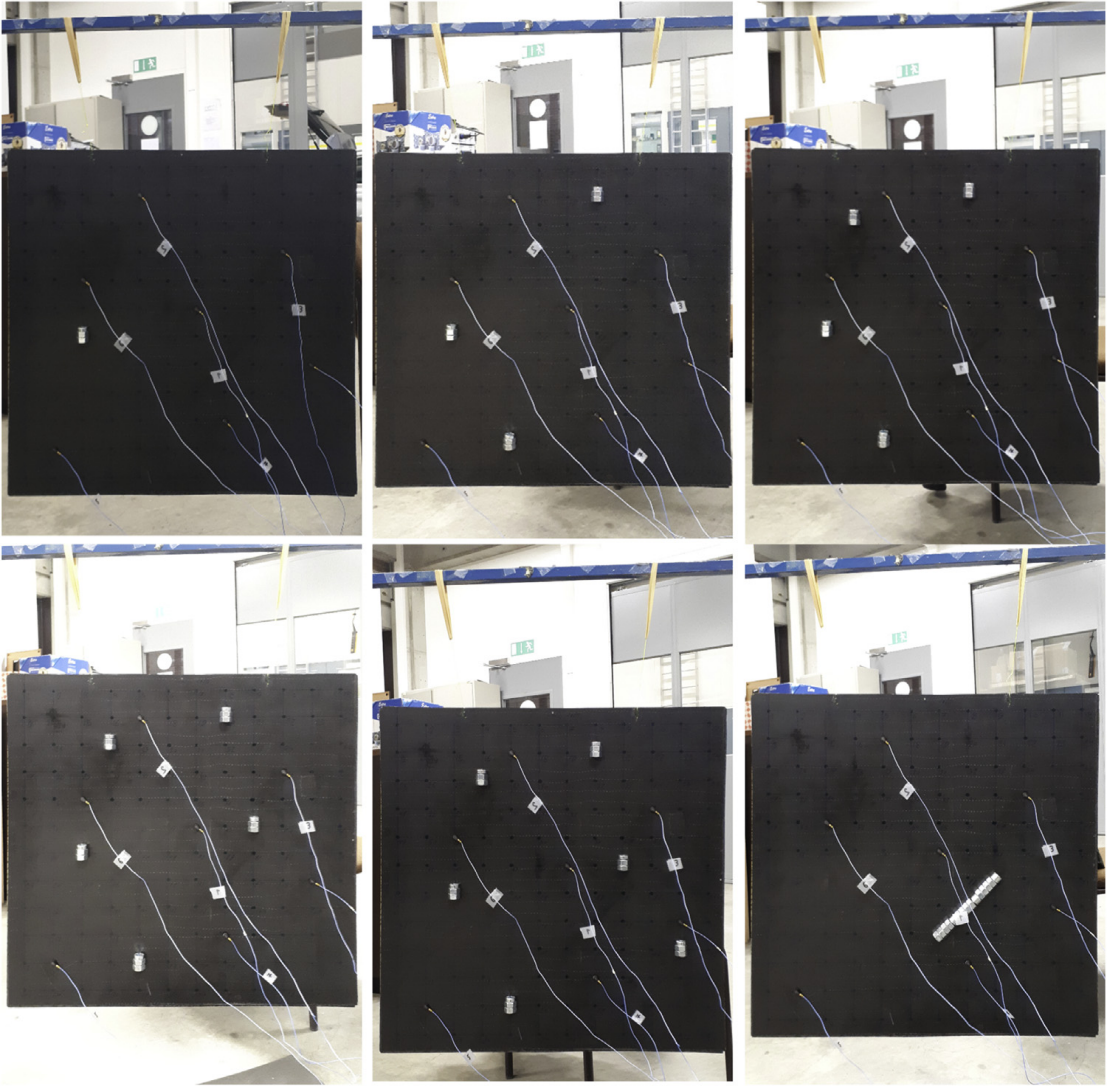
Plate hung with elastic bands, with masses added according to scenarios 1 to 6.

Additional masses are glued to the plate to reproduce the scattering effect of damage. Two types of scenarios are considered: point-like masses at a number of pre-defined nodes on the grid and an elongated mass covering several nodes. Each point mass consists of an M12 × 20 mm bolt with hexagonal socket head, assembled to 2 M12 nuts. The weight of each point mass is 55 g, which represents 2.4% of the plate mass. The elongated mass is assembled out of 3 M12 × 40 mm bolts with hexagonal socket head, and 12 M12 nuts. The weight of this assembly is 315 g, which represents 13.9% of the plate mass. A total of 6 scenarios are considered, 5 scenarios with 1, 3, 4, 5 and 6 point masses added, respectively, and 1 scenario with 1 elongated mass added. Fig. 2 depicts each of these scenarios in order.

**Fig. 3:**
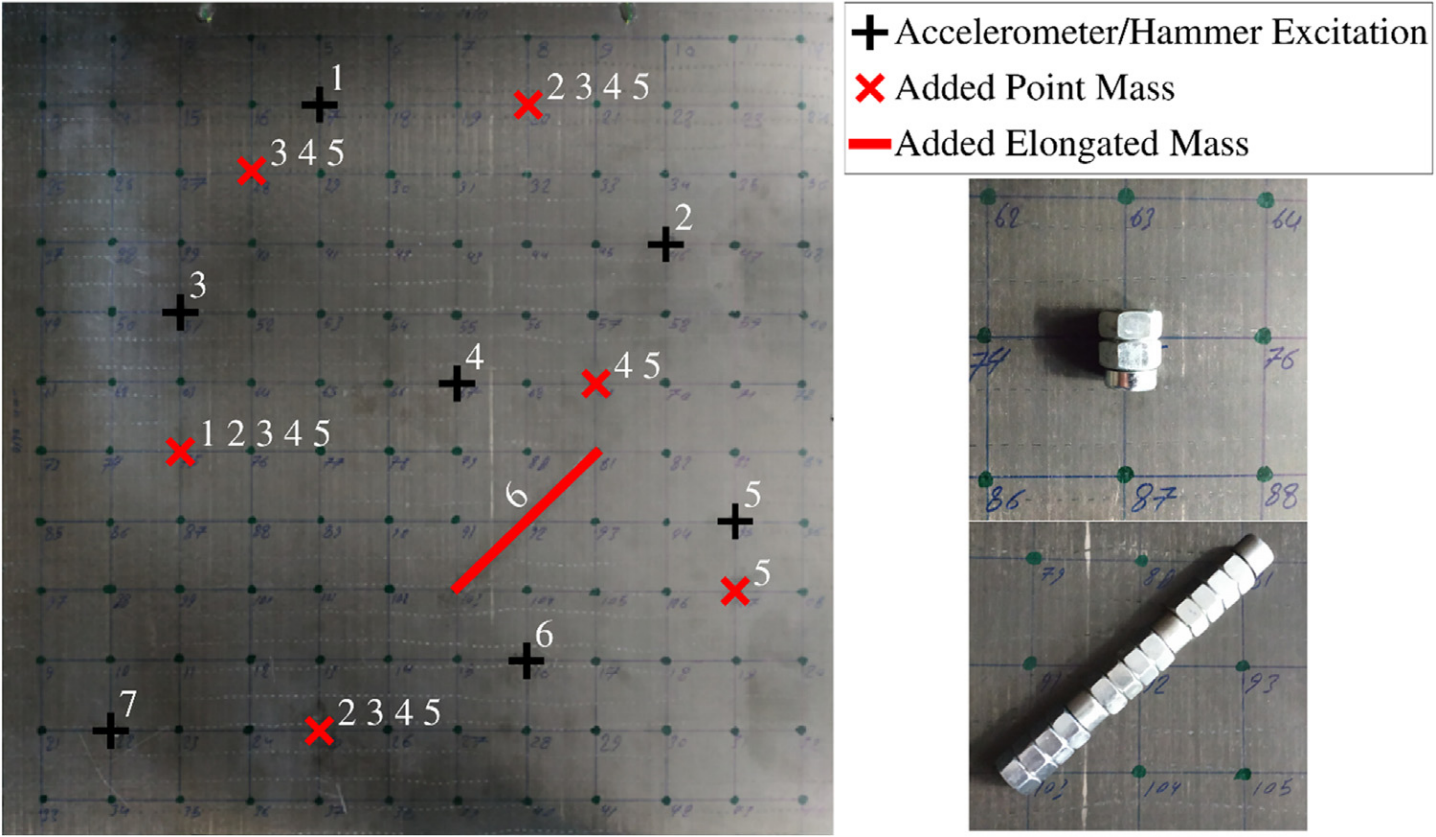
(Reproduced from [1]): Experimental setup: CFRP plate with grid of 12 × 12 nodes. The positions of the 7 accelerometers/hammer impacts are indicated in black and the positions of the added masses per scenario are indicated in red. Zoomed views are also provided of one of the 55 g point-masses and the 315 g elongated mass, as glued to the plate.

Fig. 3 summarizes the experimental setup and the inspection scenarios considered. Fig. 3 makes the 12 × 12 grid of nodes clearly visible, and symbolically indicates the positions of the 7 accelerometers (black, with numbering according to accelerometer index *j*) and of the added masses (red, with numbering according to which scenarios have a mass added at that position).

### 4.3 Data acquisition

Data was acquired according to the following procedure. First, to establish the baseline responses, the responses $T_{ij}\left(f\right)$ are measured on the plate, where the index i=1,…,144 spans all candidate damage locations, and the index j=1,…,7 spans the accelerometer locations. Then, for the subsequent inspection scenarios, the corresponding masses are glued to the plate and the altered responses $T_{ij}^{★}\left(f\right)$ are measured, where this time both the indices i=1,…,7 and j=1,…,7 span only the accelerometer locations.

Siemens Simcenter was used to collect the force and acceleration measurements and to compute the resulting FRFs. The FRFs consist of 8192 frequency bins between 0 and 1600 Hz. Measurements are repeated 5 times and averaged to increase the SNR. Table 6 lists the acquisition settings used for Impact Testing.

**Table 6:** Impact Testing acquisition settings.

**Acquisition Settings**	
Bandwidth	1600.00 Hz
Spectral lines	8192
Resolution	0.1953124 Hz
Acquisition time	5.120 s
Averages	5
**Accelerometers**	
Nominal sensitivity	100 mV/g ± 10%, individually calibrated
Input range	3.16 V
**Impact Hammer Force Cell**	
Nominal sensitivity	2.25 mV/N
Input range	158 mV
Trigger level	2.6 mV
Pretrigger	1.00 %

## Limitations

In scenario 2 (3 55 g point-masses added), some of the FRFs measured at accelerometer 1 were polluted by an abnormally large spike around 1588 Hz. In order of decreasing magnitude of the artifact, the affected FRFs are: excitation at accelerometer 2 to measurement at accelerometer 1, excitation at accelerometer 1 to measurement at accelerometer 1, excitation at accelerometer 6 to measurement at accelerometer 1, excitation at accelerometer 7 to measurement at accelerometer 1, and excitation at accelerometer 4 to measurement at accelerometer 1.

## Ethics Statement

The authors have read and follow the ethical requirements for publication in Data in Brief. This work does not involve human subjects, animal experiments or any data collected from social media platforms.

## CRediT Author Statement

**Nathan Dwek**: Software, Validation, Data Curation, Visualization, Writing – Original Draft. **Dennis Janssens**: Methodology, Investigation, Data Curation. **Vasileios Dimopoulos**: Conceptualization, Methodology. **Matteo Kirchner**: Validation, Writing – Review and Editing, Supervision. **Elke Deckers**: Conceptualization, Supervision, Validation, Writing – Review and Editing, Project Administration, Funding Acquisition. **Frank Naets**: Supervision, Writing – Review and Editing, Funding Acquisition.

## Data Availability

ZenodoLMSD 2021 Dataset for Damage Identification in Plates (Original data).
